# Supplementary material for: A Global Screen for Assembly State Changes of the Mitotic Proteome by SEC-SWATH-MS
Source: Cell Syst. 2020 Feb 26;10(2):133–155.e6. doi: 10.1016/j.cels.2020.01.001 (PMC7042714; doi:10.1016/j.cels.2020.01.001)
Supplement: Data S1. SEC-SWATH-MS Protein Chromatograms, Related to Figure 1 [file mmc6.zip › SECchrom_O75643_U520_HUMAN_SNRNP200_ASCC3L1_HELIC2_KIAA0788.pdf]

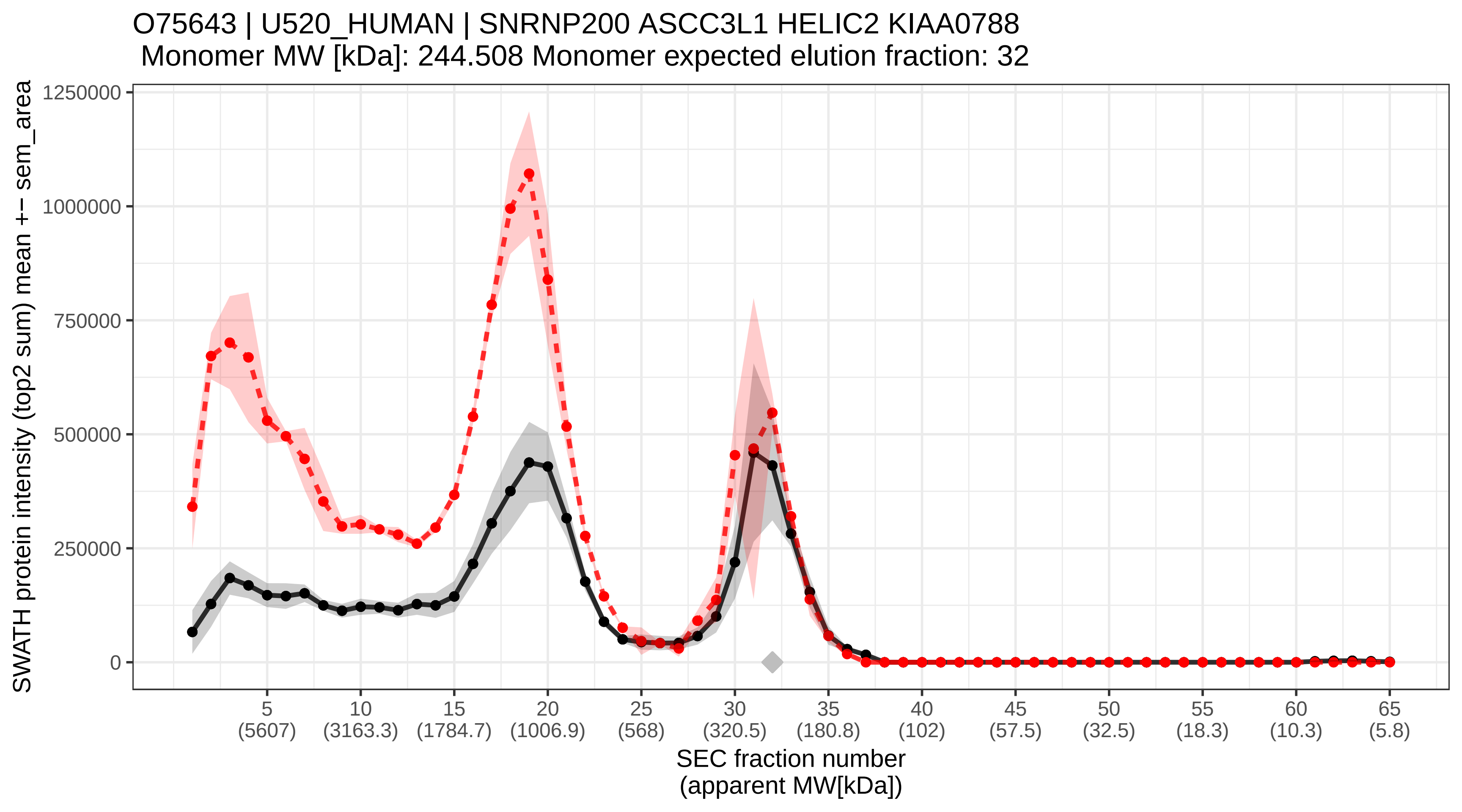

O75643 | U520_HUMAN | SNRNP200 ASCC3L1 HELIC2 KIAA0788
Monomer MW [kDa]: 244.508 Monomer expected elution fraction: 32
SWATH protein intensity (top2 sum) mean +− sem_area
1250000
1000000
750000
500000
250000
0
5
(5607)
10
(3163.3)
15
(1784.7)
20
(1006.9)
25
(568)
30
(320.5)
35
(180.8)
40
(102)
45
(57.5)
50
(32.5)
55
(18.3)
60
(10.3)
65
(5.8)
SEC fraction number
(apparent MW[kDa])
